# COVID-19 Vaccination Uptake and Hesitancy in a National Sample of Australian Gay and Bisexual Men

**DOI:** 10.1007/s10461-022-03603-x

**Published:** 2022-01-31

**Authors:** Martin Holt, James MacGibbon, Benjamin Bavinton, Timothy Broady, Shawn Clackett, Jeanne Ellard, Johann Kolstee, Angus Molyneux, Dean Murphy, Cherie Power, John de Wit

**Affiliations:** 1 Centre for Social Research in Health, UNSW Sydney, Sydney, NSW Australia; 2 The Kirby Institute, UNSW Sydney, Sydney, Australia; 3 New South Wales Ministry of Health, Sydney, Australia; 4 Australian Research Centre in Sex, Health and Society, La Trobe University, Melbourne, Australia; 5 ACON, Sydney, Australia; 6 Department of Interdisciplinary Social Science, Utrecht University, Utrecht, Netherlands

**Keywords:** Attitudes, Australia, COVID-19, Gay and bisexual men, Hesitancy, Vaccination

## Abstract

Minority groups may face additional barriers to vaccination. In April–June 2021, we assessed the level of COVID-19 vaccination and willingness to be vaccinated in a national, online survey of 1280 gay and bisexual men in Australia. Over a quarter of the sample (28.0%) had been partially or fully vaccinated, and 80.0% of the unvaccinated were willing to be vaccinated. Vaccination was independently associated with older age, being university educated, and HIV status (with HIV-positive participants being more likely and untested participants less likely to be vaccinated). Willingness to be vaccinated was independently associated with living in a capital city and being university educated. Those who had lost income or their job due to COVID-19 were less willing to be vaccinated. Our results suggest encouraging COVID-19 vaccination among those with lower levels of health literacy and supporting those who have experienced financial stress because of the pandemic.

## Resumen

Los grupos minoritarios pueden enfrentar barreras adicionales accediendo a una vacuna. En abril-junio de 2021, evaluamos el nivel de vacunación contra el COVID-19 y la disposición a la vacuna utilizando datos de una encuesta nacional en línea de 1280 hombres gays y bisexuales en Australia. El 28% de los participantes habían sido vacunados parcial o totalmente, y el 80% de los no vacunados estaban dispuestos a vacunarse. La vacunación se asoció de forma independiente con participantes de mayor edad, con educación universitaria y su estado de VIH (los participantes VIH positivos tenían más probabilidades que los participantes sin prueba del VIH de ser vacunados). La disposición a favor de ser vacunados se asoció de manera independiente con vivir en una ciudad capital y tener estudios universitarios. Aquellos que habían perdido ingresos o su trabajo debido al COVID-19 estaban menos dispuestos a vacunarse. Nuestros hallazgos sugieren que es importante promover la vacunación contra el COVID-19 entre personas que tienen menos información en temas de salud y apoyar a quienes han sufrido estrés financiero debido a la pandemia.

## Introduction

Australia had an initially successful response to COVID-19, focused on strict border controls and public health restrictions including ‘lockdowns’ in affected jurisdictions, with the aim of minimising case numbers and community transmission. By the end of July 2021, for example, the total number of cases of COVID-19 cases was fewer than 34,000 nationally [1], equivalent to 1300 cases per million people, compared with over 45,000 per million in Germany, 86,000 per million in the United Kingdom and 105,000 per million in the United States [2]. However, outbreaks continued to occur, triggering the reimposition of restrictions and lockdowns. This generated intense public debate about Australia’s COVID-19 vaccination program, which proceeded relatively slowly compared with other countries. For example, as of July 2021, it was estimated that only 32% of the Australian population had received one COVID-19 vaccine dose, compared with 61% in Germany, 69% in the United Kingdom and 57% in the United States [3].

The COVID-19 vaccine rollout in Australia was slow to get started due to a limited supply of available vaccines, changing guidelines about which vaccines were recommended for different population groups, and vaccine hesitancy [4]. Nationally representative surveys of Australian adults found that willingness to be vaccinated ranged from 55 to 73% in April 2020 to June 2021 [5–7]. Older, higher income participants were more willing to be vaccinated, while those living in socioeconomically disadvantaged areas were more hesitant. Cross-sectional surveys conducted in July–November 2020 in Australia found higher levels of willingness to be vaccinated (70–90%), associated with older age, and higher levels of education and health literacy [8–10]. An international review conducted in 2021 suggested willingness to be vaccinated ranged widely, from 24% in Kuwait to 97% in Ecuador [11].

Australia’s COVID-19 vaccination program, which aims to provide vaccines to all eligible people for free, prioritises frontline workers and people in aged care, then older adults, people with underlying medical conditions, and finally younger people [4, 12]. Aboriginal and Torres Strait Islander people were the main minority group initially specified in the vaccination program. Due to an increased risk of developing severe COVID-19 disease [4], HIV-positive people were prioritised for COVID-19 vaccination in the phase targeting people with underlying medical conditions. In Australia, the majority of people living with HIV are gay and bisexual men (GBM) [13]. Given trust in government may affect willingness to be vaccinated, and minority populations (including gender and sexual minorities) may have less trust in government services than the general population [9, 14–16], we believe there is a case for assessing vaccine readiness and uptake in minority groups, including GBM.

While there are a range of social and structural influences that may encourage GBM to seek COVID-19 vaccination, there are others that may foster hesitancy. GBM may experience poorer health and greater challenges accessing health care than the general population, particularly if they experience stigma and discrimination related to sexuality or HIV [14, 17, 18]. Conversely, GBM may attend health services more frequently than heterosexual people, to attend for HIV and sexual health screening, seek prescriptions for pre-exposure prophylaxis (PrEP), or attend HIV care appointments [19, 20]. This may increase the opportunity for vaccination. US research has found that GBM were aware of the risks of COVID-19 transmission, but continued to meet more social contacts and have more sex partners than heterosexual men during the pandemic [21]. The same study showed that GBM compensated for these risks by engaging in higher levels of protective behaviour (distancing and mask wearing) than heterosexual men, and suggested GBM would be motivated to seek vaccination to protect themselves and other people. Another US study that included GBM echoed these findings, showing that mistrust of medicine and concerns about COVID vaccines increased hesitancy, while altruism was associated with increased willingness to be vaccinated [16].

In April–June 2021, we surveyed Australian GBM and assessed factors associated with COVID-19 vaccination and willingness to be vaccinated in an ongoing study of attitudes to HIV prevention. Our aim was to see if vaccine uptake was higher or lower than expected among GBM and to identify barriers to COVID-19 vaccination among GBM that might need to be addressed during vaccine rollout. We anticipated that, as a priority group, HIV-positive men would be more likely to have been vaccinated during the early phase of vaccine rollout but were uncertain about the level of interest in vaccination among other GBM.

## Method

### Study Design and Participants

Data were collected as part of the PrEPARE Project, a repeated, cross-sectional study of Australian GBM’s attitudes toward HIV prevention that has been conducted every two years since April 2011 [22–24]. The 2021 round was a national, online, cross-sectional survey of GBM conducted in April–June 2021 using Qualtrics software (Provo, UT). Due to the emergence of COVID-19, the study team and reference group agreed that questions about the pandemic should be added to the 2021 questionnaire, to assess the impact of the COVID-19 on participants, its potential role in disrupting HIV prevention, and to identify if GBM had specific education or health promotion needs related to COVID-19 (such as the need to encourage vaccination). The survey was promoted through community organisations, Facebook groups about HIV prevention, and paid advertisements on Facebook and the dating/hook-up app Grindr. Participants were eligible to participate in the 2021 survey if they were aged 16 years or older, lived in Australia, and identified as male or nonbinary, and gay, bisexual or queer.

Potential participants were directed to the project website where study information and a link to the survey was provided. Participants were asked to indicate consent at the start of the survey. The study was approved by the ethics committee of UNSW Sydney (HC16954) and reviewed by the community organisation ACON (2017/04).

### Measures

The questionnaire measures have been previously described [22]. We collected data on demographics including age, sexual identity, country of birth, education level, residential location, employment status, and income. Age, sexual identity, country of birth and whether GBM reside in inner city, suburban or regional areas have been previously identified as affecting access to and uptake of HIV testing, prevention and risk of HIV in Australia [24–31]. Younger men, bisexual men, GBM born in Asia and those in suburban or regional areas are less likely to access testing or use PrEP and more likely to reports risks for HIV. It was thought that these disparities might be reproduced in willingness to seek COVID-19 vaccination. Education level, employment status and income were included because socioeconomic factors can affect health-seeking behaviour, and other researchers had suggested that the impact of COVID-19 would be accentuated by socioeconomic disparities [5, 6, 8]. This was also directly assessed by asking participants, “Did you lose income or your job because of COVID-19?” (Yes/No). We also included measures of participants’ relationships, recent sexual practices, HIV status, and use of HIV pre-exposure prophylaxis (PrEP). We reasoned that GBM who engaged in more sexual activity might have heightened awareness of the risks of COVID-19, and be more motivated to seek vaccination, as US colleagues have subsequently suggested [21]. As noted above, GBM living with HIV were prioritised in the vaccination phase targeting people with underlying health conditions [4], and PrEP users are known to access services more frequently than other GBM, increasing the opportunity for vaccination [19]. Self-reported health was assessed with a single item (‘In general, do you feel your health is:’) with five response options from ‘Excellent’ to ‘Poor’ [32], on the assumption that those with poorer health might be more concerned about COVID-19 and more willing to be vaccinated. The variables included in the analyses and their categories are shown in Tables 1 and 2.

**Table 1:** Factors associated with COVID-19 vaccination among gay and bisexual men in Australia (N=1280)

	All	Unvaccinated	Vaccinated	OR (95% CI)	*p* value	AOR (95% CI)	*p* value
	N=1280 (%)	n=922 (%)	n=358 (%)				
Mean age (SD)	40.9 (14.5)	38.2 (13.4)	47.8 (14.8)	1.05 (1.04–1.06)	<0.001	1.05 (1.04–1.06)	<0.001
Sexual identity							
Gay	1034 (80.8)	727 (78.9)	307 (85.8)	Ref			
Bisexual/queer/other identity	246 (19.2)	195 (21.1)	51 (14.2)	0.62 (0.44–0.87)	0.005	0.72 (0.50–1.03)	0.075
Country of birth							
Australia	916 (71.6)	678 (73.5)	238 (66.5)	Ref			
Elsewhere	364 (28.4)	244 (26.5)	120 (33.5)	1.40 (1.08–1.82)	0.012	1.24 (0.93–1.65)	0.145
Education level							
High school	310 (24.2)	252 (27.3)	58 (16.2)	Ref			
Trade certificate	223 (17.4)	179 (19.4)	44 (12.3)	1.07 (0.69–1.65)	0.768	0.71 (0.44–1.13)	0.145
University degree	747 (58.4)	491 (53.3)	256 (71.5)	2.27 (1.64–3.13)	0.000	1.76 (1.24–2.50)	0.001
Residential location							
Capital city	897 (70.1)	636 (69.0)	261 (72.9)	Ref			
Other city, regional, rural or remote area	383 (29.9)	286 (31.0)	97 (27.1)	0.83 (0.63–1.08)	0.169		
State or territory							
New South Wales	482 (37.7)	350 (38.0)	132 (36.9)	Ref			
Victoria	318 (24.8)	229 (24.8)	89 (24.9)	1.03 (0.75–1.41)	0.852		
Queensland	209 (16.3)	144 (15.6)	65 (18.2)	1.20 (0.84–1.71)	0.321		
Other jurisdictions	271 (21.2)	199 (21.6)	72 (20.1)	0.96 (0.69–1.34)	0.809		
Employment status							
Full-time	770 (60.2)	559 (60.6)	211 (58.9)	Ref			
Part-time	196 (15.3)	147 (15.9)	49 (13.7)	0.88 (0.62–1.27)	0.499		
Student/unemployed/other	314 (24.5)	216 (23.4)	98 (27.4)	1.20 (0.90–1.60)	0.208		
Annual income (AUD)							
Less than $40,000	258 (20.2)	199 (21.6)	59 (16.5)	Ref			
$40,000−$79,999	339 (26.5)	247 (26.8)	92 (25.7)	1.26 (0.86–1.83)	0.235	1.08 (0.70–1.64)	0.735
$80,000−$120,000	318 (24.8)	216 (23.4)	102 (28.5)	1.59 (1.10–2.32)	0.015	1.27 (0.83–1.93)	0.271
More than $120,000	270 (21.1)	186 (20.2)	84 (23.5)	1.52 (1.03–2.25)	0.034	0.96 (0.62–1.51)	0.874
Prefer not to say	95 (7.4)	74 (8.0)	21 (5.9)	0.96 (0.54–1.68)	0.879	0.83 (0.47–1.47)	0.523
Lost income or job due to COVID-19							
No	953 (74.5)	675 (73.2)	278 (77.7)	Ref			
Yes	327 (25.5)	247 (26.8)	80 (22.3)	0.79 (0.59–1.05)	0.102		
Relationship status							
No partner	677 (52.9)	507 (55.0)	170 (47.5)	Ref			
Regular partner(s)	603 (47.1)	415 (45.0)	188 (52.5)	1.35 (1.06–1.73)	0.016	1.13 (0.86–1.48)	0.390
Casual sex partners (last 6 months)							
No casual partner	381 (29.8)	277 (30.0)	104 (29.1)	Ref			
Casual partner(s)	899 (70.2)	645 (70.0)	254 (70.9)	1.05 (0.80–1.37)	0.727		
Health status							
Good/very good/excellent	1142 (89.2)	822 (89.2)	320 (89.4)	Ref			
Poor/fair	138 (10.8)	100 (10.8)	38 (10.6)	0.98 (0.66–1.45)	0.905		
HIV status							
HIV-negative	1059 (82.7)	752 (81.6)	307 (85.8)	Ref			
Untested or unknown	126 (9.8)	117 (12.7)	9 (2.5)	0.19 (0.09–0.38)	<0.001	0.45 (0.22–0.93)	0.032
HIV-positive	95 (7.4)	53 (5.7)	42 (11.7)	1.94 (1.27–2.97)	0.002	3.01 (1.29–7.05)	0.011
Current use of HIV pre-exposure prophylaxis (PrEP)							
No	808 (63.1)	597 (64.8)	211 (58.9)	Ref			
Yes	472 (36.9)	325 (35.2)	147 (41.1)	1.28 (1.00–1.64)	0.053		

*OR* odds ratio, *AOR* adjusted odds ratio, *CI* confidence interval, *SD* standard deviation

**Table 2:** Factors associated with willingness to be vaccinated against COVID-19 among unvaccinated gay and bisexual men (N=921)

	All	Hesitant	Willing	OR (95% CI)	*p* value	AOR (95% CI)	*p* value
	N=921 (%)	n=183 (%)	n=738 (%)				
Mean age (SD)	38.2 (13.4)	38.4 (13.5)	38.1 (13.4)	1.00 (0.99–1.01)	0.832		
Sexual identity							
Gay	726 (78.8)	128 (69.9)	598 (81.0)	Ref			
Bisexual/queer/other identity	195 (21.2)	55 (30.1)	140 (19.0)	0.54 (0.38–0.79)	0.001	0.71 (0.47–1.07)	0.101
Country of birth							
Australia	678 (73.6)	140 (76.5)	538 (72.9)	Ref			
Elsewhere	243 (26.4)	43 (23.5)	200 (27.1)	1.21 (0.83–1.77)	0.323		
Education level							
High school	251 (27.3)	68 (37.2)	183 (24.8)	Ref			
Trade certificate	179 (19.4)	55 (30.1)	124 (16.8)	0.84 (0.55–1.28)	0.411	0.68 (0.42–1.10)	0.119
University degree	491 (53.3)	60 (32.8)	431 (58.4)	2.67 (1.81–3.93)	<0.001	1.73 (1.10–2.71)	0.018
Residential location							
Capital city	636 (69.1)	100 (54.6)	536 (72.6)				
Other city, regional, rural or remote area	285 (30.9)	83 (45.4)	202 (27.4)	0.45 (0.33–0.63)	<0.001	0.63 (0.44–0.91)	0.014
State or territory							
New South Wales	350 (38.0)	70 (38.3)	280 (37.9)	Ref			
Victoria	229 (24.9)	45 (24.6)	184 (24.9)	1.02 (0.67–1.55)	0.918		
Queensland	144 (15.6)	28 (15.3)	116 (15.7)	1.04 (0.64–1.69)	0.888		
Other jurisdictions	198 (21.5)	40 (21.9)	158 (21.4)	0.99 (0.64–1.53)	0.955		
Employment status							
Full-time	559 (60.7)	87 (47.5)	472 (64.0)	Ref			
Part-time	146 (15.9)	36 (19.7)	110 (14.9)	0.56 (0.36–0.87)	0.011	0.85 (0.48–1.49)	0.564
Student/unemployed/other	216 (23.5)	60 (32.8)	156 (21.1)	0.48 (0.33–0.70)	<0.001	0.83 (0.49–1.40)	0.481
Annual income (AUD)							
Less than $40,000	198 (21.5)	51 (27.9)	147 (19.9)	Ref			
$40,000−$79,999	247 (26.8)	51 (27.9)	196 (26.6)	1.33 (0.86–2.08)	0.203	1.10 (0.62–1.96)	0.746
$80,000−$120,000	216 (23.5)	30 (16.4)	186 (25.2)	2.15 (1.30–3.55)	0.003	1.21 (0.61–2.40)	0.578
More than $120,000	186 (20.2)	24 (13.1)	162 (22.0)	2.34 (1.37–3.99)	0.002	1.05 (0.50–2.23)	0.896
Prefer not to say	74 (8.0)	27 (14.8)	47 (6.4)	0.60 (0.34–1.07)	0.083	0.56 (0.29–1.05)	0.07
Lost income or job due to COVID-19							
No	675 (73.3)	115 (62.8)	560 (75.9)	Ref			
Yes	246 (26.7)	68 (37.2)	178 (24.1)	0.54 (0.38–0.76)	<0.001	0.62 (0.42–0.92)	0.017
Relationship status							
No partner	507 (55.0)	117 (63.9)	390 (52.8)	Ref			
Regular partner(s)	414 (45.0)	66 (36.1)	348 (47.2)	1.58 (1.13–2.21)	0.007	1.32 (0.91–1.91)	0.139
Casual sex partners (last 6 months)							
No casual partner	276 (30.0)	65 (35.5)	211 (28.6)	Ref			
Casual partner(s)	645 (70.0)	118 (64.5)	527 (71.4)	1.38 (0.98–1.94)	0.068		
Health status							
Good/very good/excellent	821 (89.1)	158 (86.3)	663 (89.8)	Ref			
Poor/fair	100 (10.9)	25 (13.7)	75 (10.2)	0.71 (0.44–1.16)	0.175		
HIV status							
HIV-negative	751 (81.5)	127 (69.4)	624 (84.6)	Ref			
Untested or unknown	117 (12.7)	38 (20.8)	79 (10.7)	0.42 (0.27–0.65)	<0.001	0.84 (0.50–1.40)	0.495
HIV-positive	53 (5.8)	18 (9.8)	35 (4.7)	0.40 (0.22–0.72)	0.002	0.55 (0.29–1.04)	0.065
Current use of HIV pre-exposure prophylaxis (PrEP)							
No	596 (64.7)	142 (77.6)	454 (61.5)	Ref			
Yes	325 (35.3)	41 (22.4)	284 (38.5)	2.17 (1.48–3.16)	<0.001	1.47 (0.96–2.24)	0.074

*OR* odds ratio, *AOR* adjusted odds ratio, *CI* confidence interval, *SD* standard deviation

There were two outcome variables: vaccination against COVID-19 and willingness to be vaccinated. Vaccination against COVID-19 was measured with the item ‘Have you been vaccinated for COVID-19 (novel coronavirus)?’ (Yes or No). The vaccination question did not discriminate between partial and full vaccination (one or two doses). Participants who had responded ‘No’ to the vaccination question were asked how willing they were to be vaccinated against COVID-19: ‘When a COVID-19 vaccine becomes available to you, how likely are you to get vaccinated?’. Response options ranged from 1=very unlikely to 5=very likely. We coded responses 1–3 as hesitant and 4–5 as willing.

### Statistical Analyses

Statistical significance was set at *p*<0.05 (two-tailed). We compared characteristics associated with the two outcome variables using logistic regression. Variables for which there were statistically significant differences at a bivariate level were then block entered into multivariate logistic regression models to identify independent relationships with COVID-19 vaccination and willingness to be vaccinated. We report unadjusted and adjusted odds ratios (OR and AOR) and 95% confidence intervals (CI). Analyses were conducted using Stata version 16.1 (StataCorp, College Station, TX).

## Results

A total of 1280 participants completed the questionnaire. Participant characteristics are shown in Table 1. The mean age was 41 years (standard deviation 14.5 years). Most identified as gay (80.8%), were Australian born (71.6%), university educated (58.4%), and lived in the capital city of their state or territory (70.1%). Participants were most likely to reside in the states of New South Wales (37.7%), Victoria (24.8%), and Queensland (16.3%). Most participants were in full-time employment (60.2%), although one quarter (25.5%) had lost income or their job due to COVID-19. Nearly half the participants (47.1%) reported a regular sex partner (or partners) while a majority (70.2%) had had casual partners in the previous six months. One in ten participants (10.8%) reported ‘poor’ or ‘fair’ health. Most participants were HIV-negative (82.7%), with smaller proportions of untested (9.8%) and HIV-positive (7.4%) participants. Over one third of participants (36.9%) was taking PrEP at the time of the survey.

Over a quarter of the sample (358/1,280, 28.0%) reported being vaccinated against COVID-19. Of the unvaccinated participants (n=921), most indicated they were ‘very likely’ (67.1%) or ‘likely’ (13.0%) to be vaccinated when a vaccine became available to them. A minority indicated they were ‘somewhat’ (3.6%) or ‘very unlikely’ (6.5%) to be vaccinated, and the remaining proportion (9.8%) responded ‘neutral/don’t know’. We classified 80.1% of unvaccinated participants as willing to be vaccinated (n=738), and 19.9% as hesitant (n=183). One participant did not respond to this question and was excluded from the analysis.

Table 1 shows sample characteristics and factors associated with vaccination against COVID-19. Bivariate statistical differences were observed between the groups by age, sexual identity, country of birth, education level, income, relationship status and HIV status. The multivariate analysis showed that vaccination against COVID-19 was independently associated with older age (AOR=1.05, 95% CI=1.04–1.06), being university educated (AOR=1.76, 95% CI=1.24–2.50), and HIV status. Participants who were untested or did not know their HIV status were less likely to be vaccinated than HIV-negative participants (AOR=0.45, 95% CI=0.22–0.93), while HIV-positive men were more likely to be vaccinated than HIV-negative participants (AOR=3.01, 95% CI=1.29–7.05).

Table 2 shows the sample characteristics of unvaccinated participants and factors associated with willingness to be vaccinated. Bivariate statistical differences were observed between the groups by sexual identity, education level, residential location, employment status, income, loss of income due to COVID-19, relationship status, HIV status, and PrEP use. The multivariate analysis showed that willingness to be vaccinated against COVID-19 was independently associated with being university educated (AOR=1.73, 95% CI=1.10–2.71). Participants who lived outside of capital cities (AOR=0.63, 95% CI=0.44–0.91) and those who had lost income or their job due to COVID-19 were less willing to be vaccinated (AOR=0.62, 95% CI=0.42–0.92).

## Discussion

In the second quarter of 2021 we assessed the uptake of COVID-19 vaccination and willingness to be vaccinated among gay and bisexual men across Australia. Over a quarter of the sample (28%) indicated they had been vaccinated (including both partial and full vaccinations). This was a slightly higher but not noticeably different level of vaccination than that observed in the general population at the time [3]. Willingness to be vaccinated among unvaccinated participants (at 80%) was higher than that found in representative general population surveys [5–7], but similar to that found in cross-sectional surveys [8–10]. Overall this suggests that historical factors that may have encouraged hesitancy among GBM, such as experiences of stigma or discrimination [17, 18], or a distrust of the government [14], did not inhibit vaccination in this sample.

We found that being vaccinated against COVID-19 was independently associated with older age and being HIV positive, both of which are consistent with the prioritisation of COVID-19 vaccinations in Australia [4, 12]. Having a university degree was also associated with vaccination, aligning with research suggesting greater health literacy (and higher socioeconomic status) may contribute to seeking vaccination [5, 6, 8, 10]. GBM with lower levels of education may need additional encouragement and support to get vaccinated. The association of university education with vaccination may also reflect that some professionals (e.g. health professionals) were prioritised in the early stages of vaccine rollout. GBM who had never been tested for HIV were less likely to be vaccinated than previously tested participants, which may indicate a barrier to vaccination among those less comfortable attending health services. We note that the majority of Australian GBM (>70%) get tested for HIV at least once a year, while those using PrEP are often tested multiple times (when attending for prescriptions), which suggest repeated opportunities to discuss vaccination [19]. We did not find that recent levels of sexual activity were associated with having been vaccinated as has been suggested by others [21], although at the time we conducted the survey many of our participants would not have been eligible for vaccination, even if they had wanted to be vaccinated. Poor health status was also not associated with vaccination in the sample, but again only some health conditions were prioritised in the early vaccine rollout [4, 12].

Willingness to be vaccinated was higher among capital city residents, which aligns with previous research and reflects the location of most COVID-19 cases in Australia [9]. A higher education level was associated with likelihood of vaccination, aligning with general population surveys [8, 10], while those who had lost income or employment because of COVID-19 (a quarter of the sample) were less willing to be vaccinated. The latter result is concerning, suggesting the financial impact of COVID-19 may have created an additional barrier to vaccine rollout. Financial worries as a result of the pandemic have been identified as contributing to depression, anxiety and stress among Australian adults [33]. This suggests that organisations that work with GBM should ensure there are referral pathways to financial support and counselling when promoting COVID-19 vaccination. It has been suggested by others that GBM who continue to be sexually active during COVID-19 may be more willing to be vaccinated [21]. We did not find an independent association between sexual activity and willingness to be vaccinated, although GBM in relationships and those with recent casual sex partners did appear more willing to be vaccinated at a bivariate level.

Our findings have limitations. The primary purpose of our study was to monitor attitudes to HIV prevention among GBM and assessing the impact of COVID-19 was a secondary focus. We therefore did not collect a broader range of data that might help explain motivations for COVID-19 vaccination. For example, we did not collect participants’ occupations, and therefore could not identify frontline workers who were prioritised in the first phase of vaccine rollout, nor did we assess concerns about vaccination, such as fear of side effects [7]. The self-selected, cross-sectional sample is unlikely to be representative of GBM in Australia, which would feature a broader age range, more bisexual men, and more residents from regional areas [34], but it is aligned with samples of GBM who may be at greater risk of HIV [13, 19].

## Conclusions

The level of COVID-19 vaccine uptake and willingness we found among Australian GBM in the middle of 2021 was similar to general population samples, with increased levels of uptake among those prioritised in the vaccine rollout (such as older people and people living with HIV). We identified vaccine hesitancy among GBM with lower levels of education and those whose income or employment had been affected by COVID-19. These groups should be supported and engaged to sustain vaccine rollout across all populations, including GBM.

## Data Availability

Anonymised dataset available on request.

## Code Availability

Syntax for analysis available on request.
